# A 16-year overview of vasectomy and vasectomy reversal in the United Kingdom

**DOI:** 10.1016/j.xagr.2022.100105

**Published:** 2022-09-18

**Authors:** Sandra Wydera, Amanda Wilson

**Affiliations:** Division of Psychology, Institute for Psychological Sciences, De Montfort University, Leicester, United Kingdom

**Keywords:** descriptive statistics, United Kingdom, vasectomy, vasectomy reversal

## Abstract

**BACKGROUND:**

There is no current research to explain the trends in vasectomy and vasectomy reversals in the United Kingdom, leaving little understanding of the social phenomena involved.

**OBJECTIVE:**

This study aimed to provide an overview of vasectomy as a main method (or primary method) of contraception and vasectomy reversals among men in the United Kingdom aged >16 years.

**STUDY DESIGN:**

Descriptive statistics were generated from the National Health Service's publicly accessible datasets from 2004/2005 to 2019/2020, including data from 1,621,105 men.

**RESULTS:**

Although men are increasing their use of contraceptive services, the number of men having vasectomies and vasectomy reversals has decreased significantly.

**CONCLUSION:**

The researchers propose that more rigorous techniques for capturing data are required to understand vasectomy in the United Kingdom. There is also an urgent need for research on vasectomy in the United Kingdom and globally, not only to explain the behavioral trends found in this study, but also to explain the global trend.

**AJOG Global Reports at a Glance**

**Why was this study conducted?**

There is an absence of research on prevalence of vasectomy in the United Kingdom from 2006 onward, leaving little understanding of the current trends in the use of this contraceptive method.

**Key findings**

Although the number of men using contraceptive services has increased in the last 16 years, vasectomies and vasectomy reversals have declined significantly in the United Kingdom, by 62.23% and 81.2%, respectively.

**What does this add to what is known?**

Women are bearing the burden of contraception, and the decline in vasectomies and reversals observed in the United Kingdom is also occurring in other developed countries, with no research to explain this global trend. The researchers therefore make an urgent call for research on vasectomy and vasectomy reversal on a global scale.

## Introduction

Few studies in the United Kingdom have investigated vasectomy as a family planning method, and there is a lack of interventions to evaluate the effectiveness of programs that aim to increase vasectomy.^1^ Previous research has focused solely on urologic implications.^2^ There has also been research on the effectiveness of different methods of vasectomy,^3^ the failure rate of vasectomy,^4^ and difficulties in reversing vasectomy or extracting fertile sperm for couples who decide they want a child or to start a second family.^5^ Furthermore, research in the United Kingdom suggests that most urologists do not have specialized training required to counsel couples on the various forms of starting a family if the male partner has had a vasectomy.^6^ Vasectomy procedures continue to have low uptake, and there is an absence of research on prevalence of vasectomy in the United Kingdom from 2006 onward.^7^ With little research to guide the study, and the absence of United Kingdom research since 2006, the researchers aimed to conduct an exploratory project reviewing the publicly available data on vasectomy and vasectomy reversal in the United Kingdom over the last 16 years. On the basis of the researchers’ previous work on men and family planning in the United Kingdom, which showed that men in the United Kingdom are less likely to take contraceptive responsibility, it was hypothesized that vasectomy and vasectomy reversal rates would continue to decline over time.

## Materials and Methods

The data for this study were obtained from National Health Service (NHS) Contraceptive Services, NHS Contraceptive Service community contraceptive clinics, and Sexual and Reproductive Health Services statistical tables retrieved from NHS Digital (https://digital.nhs.uk/). NHS Digital is the national organization conducting census surveys on healthcare, the data from which are made publicly available. The researchers used the publicly available datasets from the last 16 years to create graphs that show trends in vasectomy and reversals. The analysis was conducted by the research team by downloading each year's dataset to extract and combine the 16 individual datasets downloaded from NHS Digital into one large dataset from April 1, 2004 to March 31, 2020 (datasets 2004/2005–2019/2020). For the purpose of this study, variables relevant only to male attendees were isolated and moved to a new SPSS dataset (IBM SPSS 26.0) created by the research team to enable descriptive data analysis. Data for analysis included information about the number of attendees, their age, the main method of contraception (ie, that reported by the attendee as the primary form of contraception; available choices included condoms and vasectomy), the total number of vasectomies and reversals conducted each year, and the age of attendees at the time of the vasectomy procedure. Data on male attendees’ age excluded those aged <18 years. The tables provide summary data on men in the United Kingdom, which were collected by contraceptive service providers in family planning clinics and clinics run by voluntary organizations. The census excludes data from outpatient clinics. However, the NHS is a public, tax-funded healthcare system, with the ratio of private to public spending being 17:83.^8^ Most NHS services provide vasectomy free of charge, with general practitioners and contraceptive clinics providing information on waitlists and referrals.^9^ In total, 1,621,105 individuals’ data were analyzed (2019/2020 period excluded because of missing data). Because the data are publicly available and this study was a secondary analysis, ethical approval was not required by the research team.

## Results

### General attendance, age, and main method of contraception

**Figure 1:**
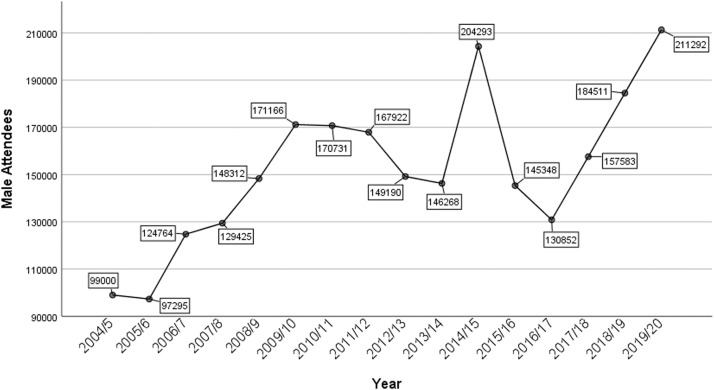
Number of male attendees of contraceptive services by year

In the last 16 years, the total number of contraceptive services attendees equaled 36,380,257, and only 5.27% of them were male (1,917,580). The number of male attendees increased from 97,000 in 2005/2006 to 171,000 in 2009/2010 and started to decrease until reaching 146,000 in 2013/2014. This number spiked in 2014/2015 (204,000), then decreased for another 2 years, after which it steadily increased from 2016/2017 to 2019/2020, reaching 211,000 (Figure 1).

Wydera. Sixteen-year overview of vasectomy in the United Kingdom. Am J Obstet Gynecol Glob Rep 2022.

**Figure 2:**
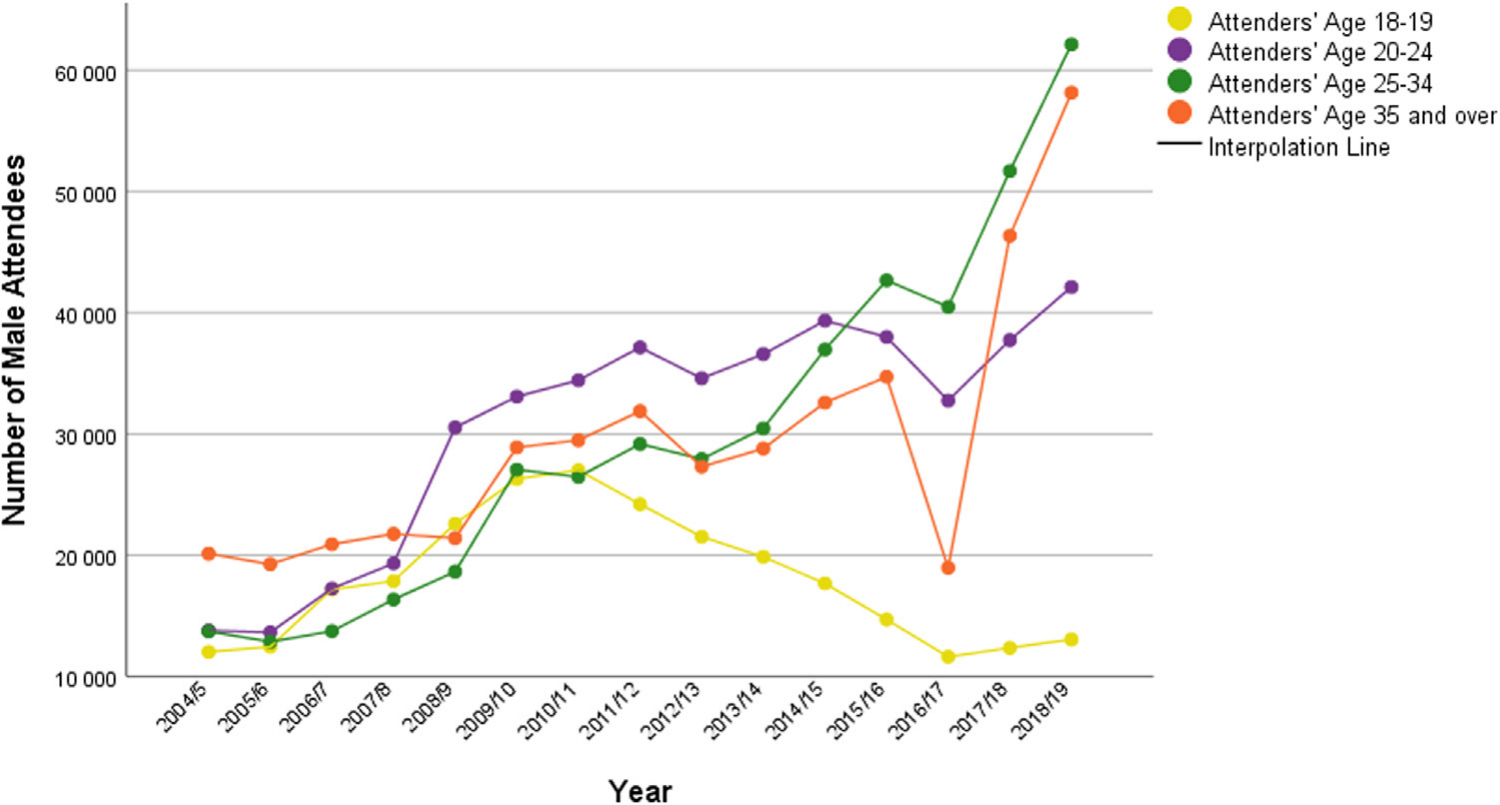
Number of male attendees by age and year

The analysis of age revealed that 14.09% of male attendees were between 18 and 19 years old, 24% were aged 20 to 24 years, 23.48% were aged 25 to 34 years, and 22.97% were aged ≥35 years. The most prevalent age group undergoing vasectomy procedures were 35 to 39-year-olds (29.76%), followed by 40 to 44-year-olds (24.54%). However, the dataset from 2018/19 did not include details of male attendees’ age. It was impossible to determine the third most prevalent age because since 2013/14, those aged 45 to 49 and ≥50 years were merged together into the ≥45-year-old group. Therefore, the percentages mentioned were calculated using data from 14 years (2004/5–2018/19). Figure 2 illustrates how the number of male attendees in the different age groups changed by year.

Wydera. Sixteen-year overview of vasectomy in the United Kingdom. Am J Obstet Gynecol Glob Rep 2022.

The number of attendees aged 20 to 24 years increased from 19,300 to 30,500 in 2008/2009 and was continuously increasing until 2011/2012. In addition, the number of attendees aged 18 to 19 years was decreasing steadily between 2010/2011 and 2016/2017, with the difference between those years totaling 15,000. Moreover, in 2016/17 there was a notable decrease in the number of attendees aged ≥35 years. In 2017/18 and 2018/2019, the number of male attendees increased.

**Figure 3:**
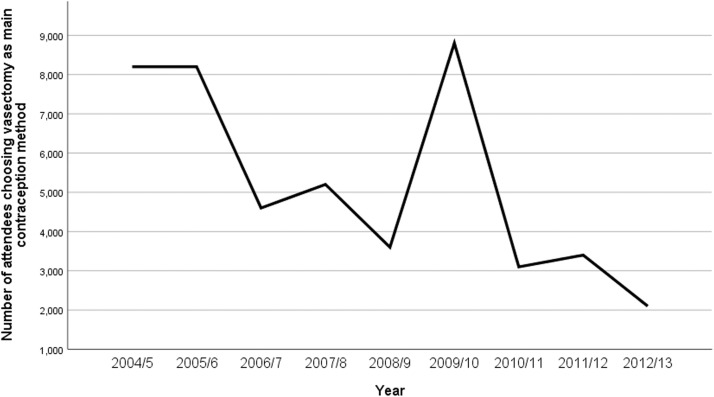
Number of male attendees choosing vasectomy as main contraceptive method

Of the men attending contraceptive services, 95% reported condoms as the main method of contraception, and the remaining 5% reported vasectomy. Moreover, the number of attendees choosing vasectomy as the main method of contraception was decreasing by year, with the exception of the year 2009/2010 when it reached approximately 8800 attendees. The data from 2013/14 onward recorded only the number of attendees choosing condoms as their main method of contraception. Therefore, Figure 3 illustrates trends in vasectomy as the main contraception method from 2004/5 to 2012/13.

Wydera. Sixteen-year overview of vasectomy in the United Kingdom. Am J Obstet Gynecol Glob Rep 2022.

### Vasectomy

**Figure 4:**
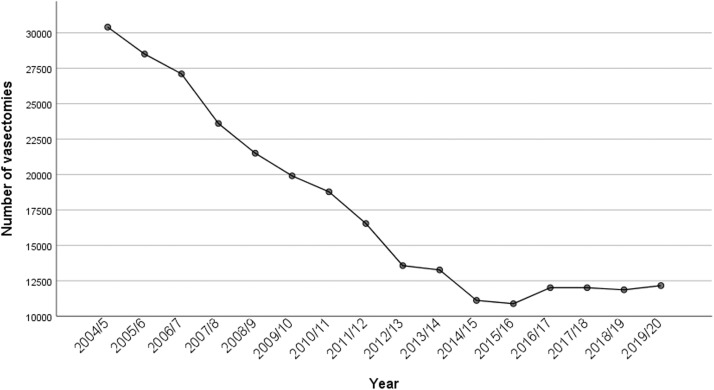
Number of performed vasectomies by year

The number of conducted vasectomies decreased continuously by year. As shown in Figure 4, in 2004/2005 approximately 30,400 vasectomies were conducted, whereas in 2015/2016 the number of conducted vasectomies totaled 10,880. This accounts for a 62.21% decrease in the number of vasectomies. Since 2016/17, the number of vasectomies was increasing slightly but did not reach >12,200 individuals (Figure 4).

Wydera. Sixteen-year overview of vasectomy in the United Kingdom. Am J Obstet Gynecol Glob Rep 2022.

**Figure 5:**
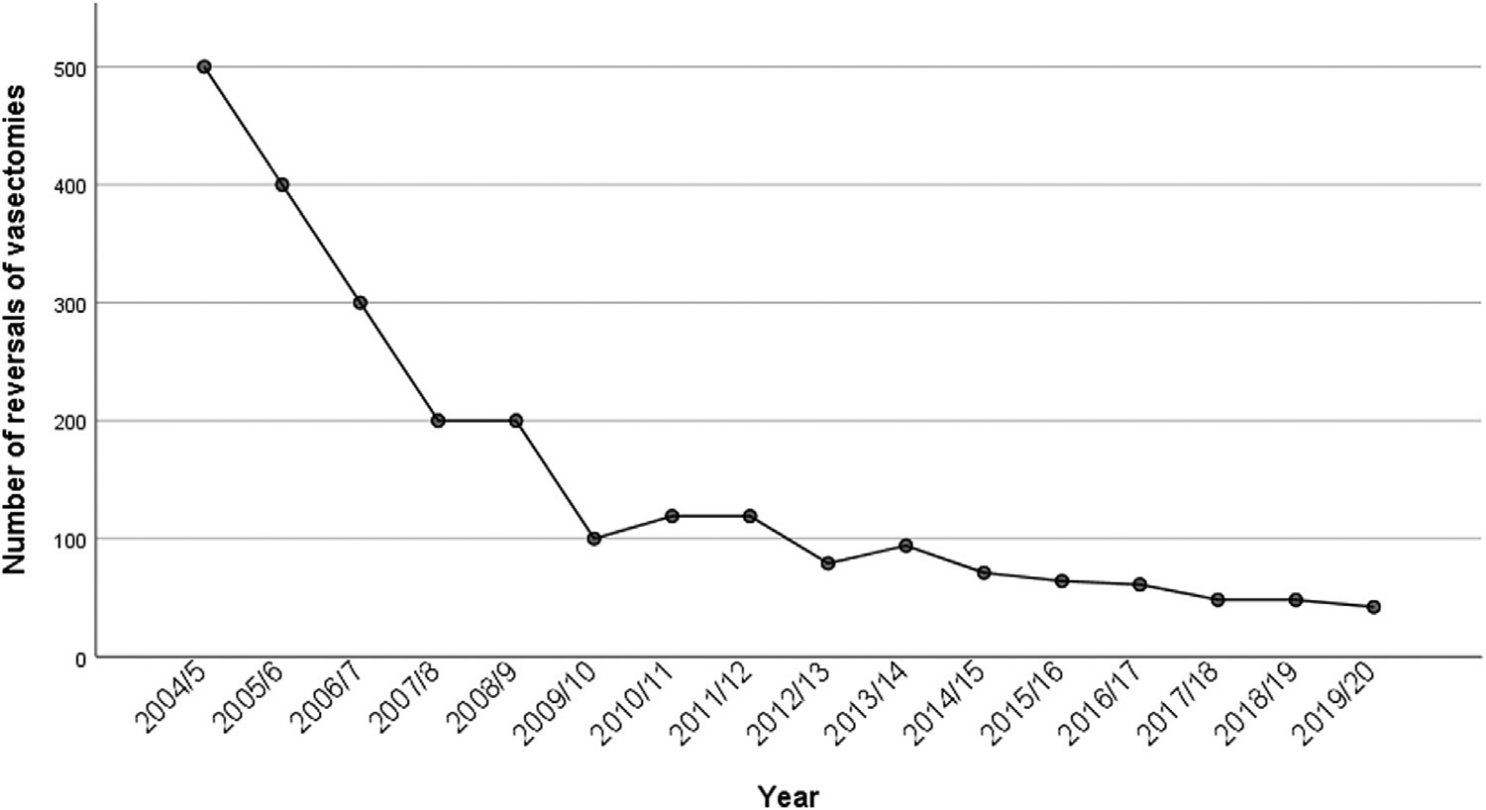
Number of vasectomy reversals conducted by year

The number of vasectomy reversals was also decreasing by year (Figure 5). In 2004/2005, approximately 500 reversals of vasectomy were conducted, whereas in 2013/2014 this number equaled 94, accounting for a 81.2% decrease in the number of vasectomy reversals (Figure 5).

Wydera. Sixteen-year overview of vasectomy in the United Kingdom. Am J Obstet Gynecol Glob Rep 2022.

## Discussion

Although the number of male attendees using contraceptive services has increased in recent years, the number of male attendees seeking vasectomy has decreased. Vasectomy rates have also declined in other high-income countries.^10^^,^^11^ This suggests a global trend, but there is little research to explain why it is occurring. There is also little research to explain the decrease of reversals over time, which may suggest better family planning practices. In addition, the decrease could be a result of the change in National Institute for Health and Care Excellence (NICE) Guidelines in 2005, which suggested that female long-acting reversible contraception was most cost-effective in reducing unintended pregnancy. There was then an initiative to increase the number of women choosing this option, for which the data show a successful increase from 2005 to 2010.^12^ The researchers hypothesize that although the number of men using contraceptive services increased from 2009 to 2010, there was no change in vasectomy procedures because they were being moved from hospital to community care with associated funding problems, which subsequently also led to the decrease in vasectomy procedures carried out by the NHS.^13^ However, without further research this trend cannot be truly understood. Other possible explanations are that the cost of reversal is usually not covered by the NHS,^9^ and private surgeons do not need to report vasectomy reversal procedures to the Department of Health^14^ overseen by the NHS Digital census. This study further highlights how women are bearing the burden of contraception and sterilization for family planning,^1^ and also the burden of unplanned or unintended pregnancy.^15^

Limitations include the datasets’ lack of consistency in recorded variables such as the age of male attendees: since 2013/2014, those aged 45 to 49 and ≥50 years were combined into the ≥45-year-old group*.* Secondly, until 2012/2013 the datasets recorded men's main contraception method as either condoms or vasectomy, but since 2013/2014 the categories changed to include other methods such as female methods. The lack of baseline and consistent measures made comparison between male and female methods impossible over a 16-year time period. In addition, this study captured the data of male attendees from contraceptive clinics and voluntary organizations, but it did not capture vasectomies provided by private providers. This is because the NHS does not require external or private providers to report data to the national census, meaning that the rates of vasectomies and reversals could be underestimated. However, the NHS census has no way to trace whether men participate in the census more than once, and thus the rates of vasectomies and reversals may be overestimated in this study. These limitations may have decreased the quality and accuracy of the collected data and prevented any advanced statistical analysis from being conducted.

## Conclusion

There is an urgent need to understand why vasectomy and vasectomy reversals are decreasing in the United Kingdom and globally to begin to address the dearth of research on why this trend is occurring. The researchers call for more rigorous methods by the NHS when collecting and reporting data on the number of men who undergo vasectomy and reversal, including the collection of data from private clinics, to better understand vasectomy trends in the United Kingdom and the involved social phenomena. Men can act as partners in family planning to improve maternal health; however, the dataset did not provide a clear picture of how men can be or are being engaged to do so.
